# Risk of SARS-CoV-2 Reinfection in Children Within the 12 Months Following Mild COVID-19: Insights From a Survey Study

**DOI:** 10.1097/INF.0000000000004233

**Published:** 2024-01-18

**Authors:** Costanza Di Chiara, Riccardo Boracchini, Anna Cantarutti, Fatima Kakkar, Andrea Oletto, Andrea Padoan, Daniele Donà, Carlo Giaquinto

**Affiliations:** * From the Department for Women’s and Children’s Health, University of Padua, Padua, Italy; † Penta – Child Health Research, Padua, Italy; ‡ Division of Biostatistics, Epidemiology and Public Health, Laboratory of Healthcare Research and Pharmacoepidemiology, Department of Statistics and Quantitative Methods, University of Milano-Bicocca, Milan, Italy; § Division of Infectious Diseases, Department of Pediatrics, CHU Sainte-Justine, Montréal, Québec, Canada; ¶ Department of Medicine-DIMED, University of Padua, Padua, Italy.

**Keywords:** SARS-CoV-2, humoral correlates of protection, COVID-19, reinfection, children

## Abstract

Understanding the correlation between immune response and protection from COVID-19 will play a pivotal role in predicting the effectiveness of vaccines in children. We studied SARS-CoV-2 reinfection risk in children 12 months post-mild COVID-19. Children under 5 years old exhibited lower reinfection risk than older infected or vaccinated siblings during 12 months postimmunization.

Previous studies have shown that antibody titers elicited by SARS-CoV-2 infection or COVID-19 vaccination are associated with protection against SARS-CoV-2 reinfection in adults.^1,2^ However, there is limited data on the protective role of anti-SARS-CoV-2 antibodies in children. With the approval of COVID-19 vaccines for infants and young children, understanding the humoral correlates of protection in the pediatric population has become highly important, as it may help optimize the timing of vaccination for this age group.

In a previous cohort study on COVID-19 family clusters, we documented an inverse correlation between the humoral response to SARS-CoV-2 and age.^3^ Specifically, it was found that children under 5 years of age exhibited higher levels of antireceptor-binding-domain (anti-RBD) IgG antibody titers compared to older individuals up to 18 months after experiencing mild SARS-CoV-2 infection.^3^

Therefore, the objective of this study was to investigate the risk of SARS-CoV-2 reinfection among children under 5 years old who had previously experienced mild COVID-19, as compared to older siblings 5–22 years old who were either previously SARS-CoV-2 infected or vaccinated.

## METHODS

A survey study was designed as a cross-sectional web-based self-administered questionnaire to assess SARS-CoV-2 reinfection during the 12 months following primary mild SARS-CoV-2 infection or COVID-19 vaccination in children and adolescents included in the COVID-19 family cluster outpatient cohort of the University Pediatric Hospital of Padova, Italy.^4^ The survey was circulated between December 23, 2022, and April 30, 2023, to 393 families who had experienced mild SARS-CoV-2 infection within their households. The severity of COVID-19 was defined according to the World Health Organization COVID-19 clinical classification.^5^

The questionnaire was emailed to parents using the REDCap platform (Vanderbilt University, Tennessee). Parents completed 1 questionnaire for each of their children 0–22 years of age. Information on children’s close contact with confirmed COVID-19 cases, occurrences of SARS-CoV-2 reinfections, and COVID-19 vaccination history were collected. Children were categorized based on age and immunization status into 4 exposure groups: (1) SARS-CoV-2-recovered participants 0–<5 years (reference group), (2) SARS-CoV-2-recovered participants ≥5–22 years, (3) naïve-vaccinated participants ≥5–22 years who received a 2-doses mRNA COVID-19 vaccination and (4) participants ≥5–22 years old with hybrid immunity defined as single-dose mRNA COVID-19 vaccination within 12 months after previous SARS-CoV-2 infection. Patients were followed up to 12 months from the index date, defined as the date of the first SARS-CoV-2 infection (groups a and b) or the date of COVID-19 vaccination (groups c and d). The follow-up was stopped if a laboratory-confirmed SARS-CoV-2 reinfection (the outcome of interest) or booster vaccination occurred (see Figure, Supplemental Digital Content 1, http://links.lww.com/INF/F380).

Sociodemographic and clinical characteristics were summarized according to numbers and percentages and median and interquartile range (IQR), as appropriate. Kaplan–Meier survival estimation was applied to assess the time free from SARS-CoV-2 reinfection in the study cohort throughout the 12 months of follow-up. Cox proportional hazard regression was used to analyze the association between exposure groups and reinfection, expressed as hazard ratio (HR) with 95% confidence interval (CI) using group a as reference. The adjustment was based on sex, the presence of at least one comorbidity, and SARS-CoV-2 lineages (pre-Omicron and/or omicron lineages).

## RESULTS

A total of 156/393 families participated in the study, resulting in a survey response rate of 40%. No differences in children’s median age [8.9 (IQR = 5.7–12.3) vs. 9.8 (IQR = 6.2–13) years, *P* = 0.12], presence of at least one child with at least one underlying condition within the family [N = 59/139 (38%) vs. 78/237 (33%), *P* = 0.31], and the median number of children included in the family [2 (IQR = 1–2) vs. 2 (IQR = 1–2) children, *P* = 0.89] were observed between responder and nonresponder families. Conversely, responder families showed a shorter time distance between the family cluster infection date and the survey compared to nonresponder families [median time = 716 (IQR = 360–777) vs. 737 (IQR = 477–794) days, *P* = 0.03] (see Table, Supplemental Digital Content 2, http://links.lww.com/INF/F380).

The study included 208 children, 68 in group a, 59 in group b, 42 in group c and 39 in group d. The median time from the index date to the survey was 741.6 days (IQR = 402.1–787.8). Among the included children, 168 (81%) experienced SARS-CoV-2 infection in the pre-Omicron era, while 40 (19%) experienced infection during the Omicron era (see Table, Supplemental Digital Content 3, http://links.lww.com/INF/F380).

**FIGURE 1.**
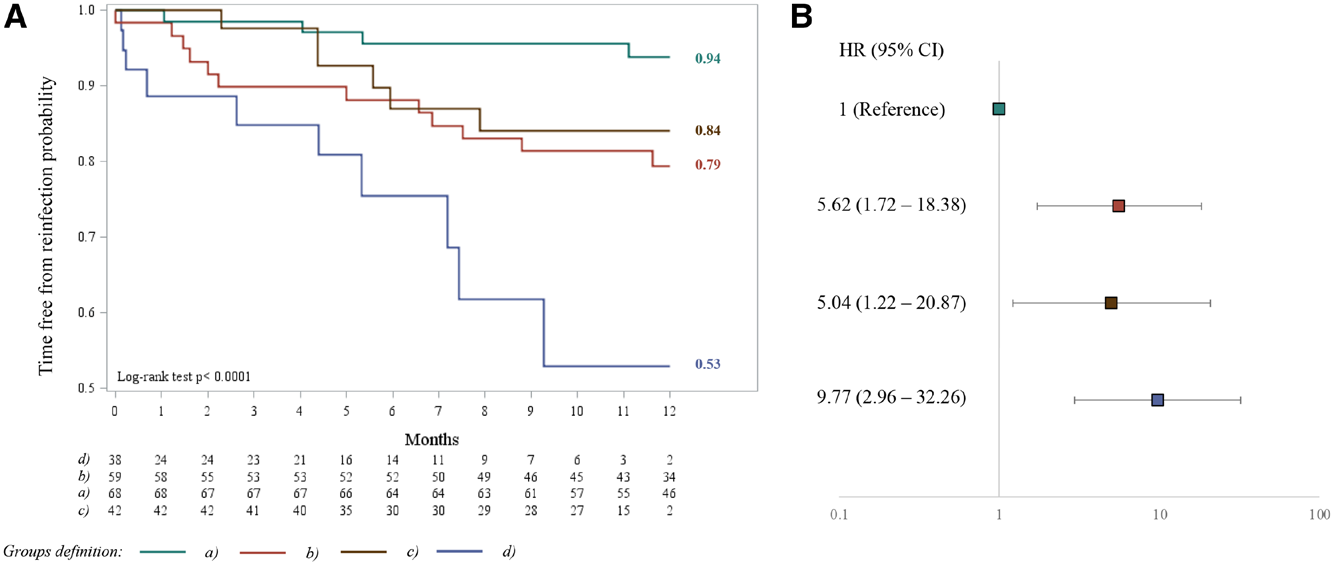
Cumulative time free from SARS-CoV-2 reinfection during the 12 months postimmunization among the exposure groups (A). Adjusted hazard ratio and 95% CI for SARS-CoV-2 reinfection in the 12 months postimmunization, using SARS-CoV-2-recovered participants 0–<5 years of age as reference group (B). Group definitions: (a) SARS-CoV-2-recovered participants 0–<5 years old (reference group), (b) SARS-CoV-2-recovered participants ≥5–22 years old, (c) naïve-vaccinated ≥5–22 years of age and (d) participants ≥5–22 years old with hybrid immunity defined as single-dose vaccination within 12 months after previous SARS-CoV-2 mild infection.

The probability of remaining free from SARS-CoV-2 reinfection was significantly higher in children <5 years old (94%) compared to older SARS-CoV-2-recovered (79%), older naïve-vaccinated (84%) and older children with single-dose hybrid immunity (53%) in the 12 months postimmunization (*P* < 0.0001) (Fig. 1A). Severe reinfections requiring hospitalization or mortality were not reported in any group. Interestingly, the probability of remaining free from reinfection was low in individuals 5–22 years of age, with hybrid immunity achieved through a single-dose vaccination after previous SARS-CoV-2 infection. Conversely, it was similar between SARS-CoV-2-recovered (79%) and naïve-vaccinated (84%) children 5–22 years of age.

Cox model analysis confirmed the increased reinfection risk in group b (HR = 5.62, 95% CI: 1.72–18.38), group c (HR = 5.04, 95% CI: 1.22–20.87) and group d (HR = 9.77, 95% CI: 2.96–32.26) compared with those SARS-CoV-2-recovered <5 years old (group a), regardless of previous SARS-CoV-2 infection in the pre- or Omicron era (Fig. 1B).

## DISCUSSION

Our findings showed that children under 5 years of age who had experienced mild SARS-CoV-2 infection had a lower probability of reinfection compared to older infected and/or vaccinated siblings up to 12 months after primary infection or COVID-19 vaccination.

Since it has been largely documented an inverse correlation between SARS-CoV-2 humoral response and age, with young children producing higher titers of SARS-CoV-2-specific antibodies compared to older individuals,^3,6,7^ our findings suggest that higher humoral responses to SARS-CoV-2 in infants and young children may provide robust protection against reinfection in the pediatric population. This hypothesis parallels previous studies showed promising results on SARS-CoV-2 humoral correlates of protection in adults.^1,2,8^

Furthermore, our study showed that a single-dose mRNA COVID-19 vaccination within 12 months after previous SARS-CoV-2 infection may provide limited protection, highlighting the importance of administering a 2-dose primary vaccination series, even in children with previous SARS-CoV-2 infection. These results are consistent with previous research that has demonstrated the superior efficacy of a 2-dose hybrid immunity in SARS-CoV-2-recovered adults.^9^

In the same cohort of COVID-19 family clusters, we have previously documented a progressive decay of anti-RBD IgG and neutralizing antibody titers over time in the pediatric population, regardless of age.^3,7^ In line with these previous findings, we observed a waning in protection against SARS-CoV-2 reinfection over time in all groups, highlighting the importance of longitudinal studies to identify a protective threshold of anti-SARS-CoV-2 antibody titers that would trigger COVID-19 vaccination.

This study has several limitations. First, due to the self-reported nature of the survey, asymptomatic undiagnosed reinfections cannot be excluded. Therefore, this study primarily focused on the reduced risk of symptomatic infections rather than asymptomatic infections, leading to a possible underestimation of the outcome. Moreover, the limited engagement of children <5 years of age in outdoor activities may have contributed to a decreased susceptibility to viral infections, including SARS-CoV-2, in this age group. Furthermore, the shorter time span from the onset of the COVID-19 family cluster to the survey completion in responder families compared to nonresponder families could potentially introduce a selection bias.

Further studies with larger sample sizes are necessary to confirm our findings and assess humoral correlates of protection in SARS-CoV-2-recovered and vaccinated children, particularly in the context of emerging viral variants. Moreover, further studies are needed to document the memory B cells’ protective role against SARS-CoV-2 reinfection in young children. Understanding the relationship between immune responses and protection from COVID-19 will be instrumental in predicting the effectiveness of vaccines in children and guiding the development of optimized vaccination timing for this population.

## ACKNOWLEDGMENTS

*The corresponding author thanks Dr. Bertilla Ranzato for her support in survey distribution. The authors thank all families who attended the COVID-19 family cluster clinic of the University Hospital of Padova and participated in the survey.*

## Supplementary Material



## References

[1] Khoury DS Schlub TE Cromer D. Correlates of protection, thresholds of protection, and immunobridging among persons with SARS-CoV-2 infection. Emerg Infect Dis. 2023;29:381–388. 36692375 10.3201/eid2902.221422 PMC9881762

[2] Perry J Osman S Wright J. Does a humoral correlate of protection exist for SARS-CoV-2? A systematic review. PLoS One. 2022;17:e0266852. 35395052 10.1371/journal.pone.0266852 PMC8993021

[3] Di Chiara C Cantarutti A Costenaro P. Long-term immune response to SARS-CoV-2 infection among children and adults after mild infection. JAMA Netw Open. 2022;5:e2221616. 35816313 10.1001/jamanetworkopen.2022.21616 PMC9280400

[4] Di Chiara C Boracchini R Sturniolo G. Clinical features of COVID-19 in italian outpatient children and adolescents during parental, delta, and omicron waves: a prospective, observational, cohort study. Front Pediatr. 2023;11:1193857. 37635788 10.3389/fped.2023.1193857 PMC10450148

[5] Clinical management; 2021. Available at: https://www.who.int/publications/i/item/WHO-2019-nCoV-clinical-2021-2.

[6] Yang HS Costa V Racine-Brzostek SE. Association of age with SARS-CoV-2 antibody response. JAMA Netw Open. 2021;4:e214302. 33749770 10.1001/jamanetworkopen.2021.4302 PMC7985726

[7] Bonfante F Costenaro P Cantarutti A. Mild SARS-CoV-2 infections and neutralizing antibody titers. Pediatrics. 2021;148:e2021052173. 34158312 10.1542/peds.2021-052173

[8] Goldblatt D Alter G Crotty S. Correlates of protection against SARS-CoV-2 infection and COVID-19 disease. Immunol Rev. 2022;310:6–26. 35661178 10.1111/imr.13091 PMC9348242

[9] Carazo S Skowronski DM Brisson M. Protection against omicron (B11529) BA2 reinfection conferred by primary omicron BA1 or pre-omicron SARS-CoV-2 infection among health-care workers with and without mRNA vaccination: a test-negative case-control study. Lancet Infect Dis. 2023;23:45–55. 36152671 10.1016/S1473-3099(22)00578-3 PMC9491856

