# Supplementary material for: Risk of SARS-CoV-2 Reinfection in Children Within the 12 Months Following Mild COVID-19: Insights From a Survey Study
Source: Pediatr Infect Dis J. 2024 Jan 18;43(4):e128–30. doi: 10.1097/INF.0000000000004233 (PMC10919262; doi:10.1097/INF.0000000000004233)
Supplement: Supplementary file 1 [file inf-43-e128-s001.docx]

***Supplemental Digital Contents***

***Supplemental Digital Content 1. Figure.*** Exposure groups’ definition and follow-up scheme.


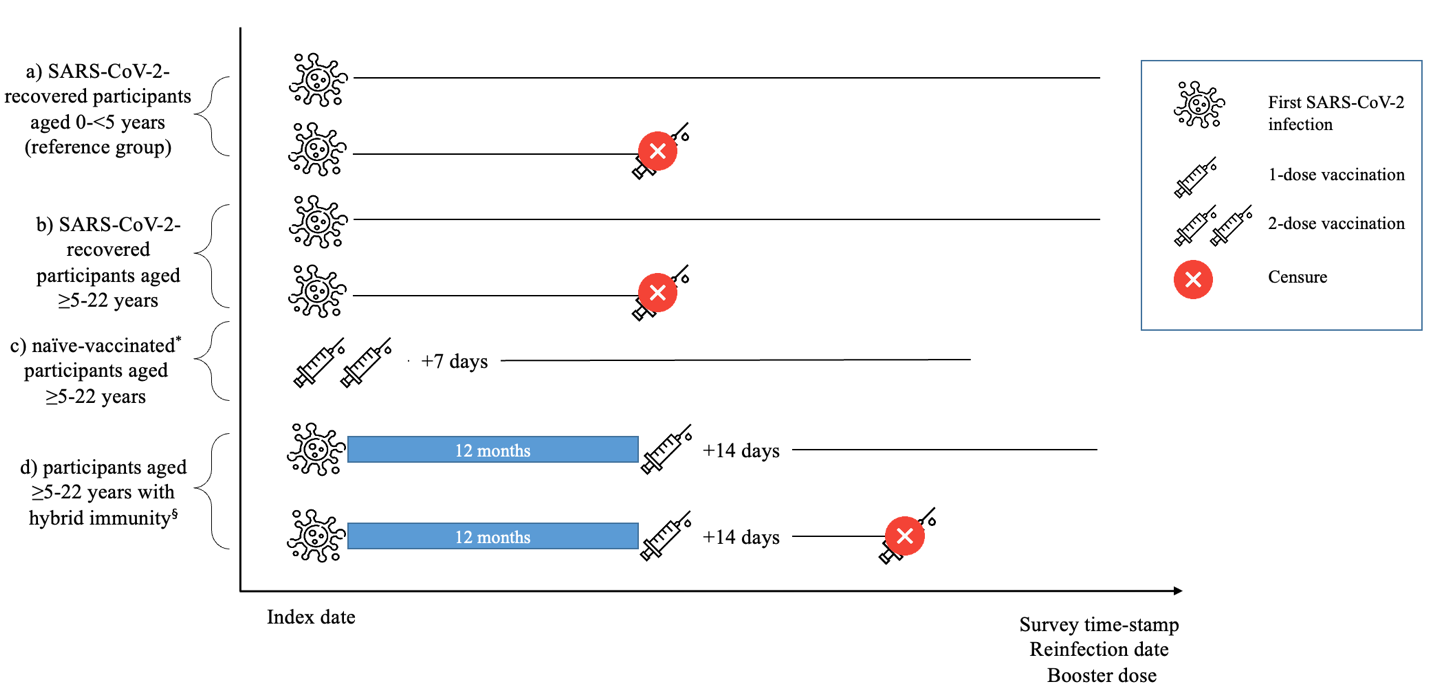


^*^naïve-vaccinated participants were defined as participants aged ≥5-22 years who received a two-doses mRNA COVID-19 vaccination

^§^ hybrid immunity was defined as single-dose mRNA COVID-19 vaccination within 12 months after previous SARS-CoV-2 infection

***Supplemental Digital Content 2. Table.*** Comparison of sociodemographic characteristics between responder and non-responder families

|  | Overall  (N = 393) | Responders  (N = 156) | Non-responders (N = 237) | p value |
| --- | --- | --- | --- | --- |
| Children's age, years, median (IQR) | 9.5 (6.0 - 12.8) | 8.9 (5.7 - 12.3) | 9.8 (6.2 – 13.0) | 0.12 |
| Number of children within the family, median (IQR) | 2 (1 - 2) | 2 (1 - 2) | 2 (1 - 2) | 0.89 |
| 1 | 155 (39.44) | 64 (41.03) | 91 (38.40) |  |
| 2 | 188 (47.84) | 70 (44.87) | 118 (49.79) |  |
| 3 | 41 (10.43) | 17 (10.90) | 24 (10.13) |  |
| 4 | 8 (2.04) | 4 (2.56) | 4 (1.69) |  |
| 5 | 1 (0.25) | 1 (0.64) | 0 (0) |  |
| Number of children  with at least one underlying  condition within the family, N (%) | | |  |  |
| 0 | 256 (65) | 97 (62) | 159 (67) | 0.31 |
| ≥1 | 137 (35) | 59 (38) | 78 (33) |  |

***Supplemental Digital Content 3. Table.*** Sociodemographic and clinical characteristics of study participants by exposure groups: Group a) SARS-CoV-2-recovered participants aged 0-<5 years (reference group), Group b) SARS-CoV-2-recovered participants aged ≥5-22 years, Group c) naïve-vaccinated participants aged ≥5-22 years, and Group d) partecipants aged ≥5-22 years with hybrid immunity.

|  | Group a  (N = 68) | Group b (N = 59) | Group c  (N = 42) | Group d (N = 39) |
| --- | --- | --- | --- | --- |
|  | N (%) | N (%) | N (%) | N (%) |
| Sex |  |  |  |  |
| Male | 40 (59) | 41 (69) | 24 (57) | 21 (54) |
| Female | 28 (41) | 18 (31) | 18 (43) | 18 (46) |
| Participants with at least one underlying condition |  |  |  |  |
| 0 | 51 (75) | 43 (73) | 37 (88) | 26 (67) |
| ≥1 | 17 (25) | 16 (27) | 5 (12) | 13 (33) |
| COVID-19 severity  (WHO clinical score)^§^ |  |  |  |  |
| Asymptomatic | 11 (16) | 15 (25) | - | 11 (28) |
| Mild | 57 (84) | 42 (71) | - | 28 (72) |
| Moderate / Severe | 0 (0) | 0 (0) | - | 0 (0) |
| Critical | 0 (0) | 0 (0) | - | 0 (0) |
| COVID-19 pandemic era  at infection^¥^ |  |  |  |  |
| Pre-omicron era | 49 (72) | 39 (67) | 8 (100) | 37 (95) |
| Omicron era | 19 (28) | 19 (33) | 0 (0) | 2 (5) |
| SARS-CoV-2 reinfection, type of symptoms |  |  |  |  |
| Fatigue | 1 (25) | 7 (58.33) | 2 (33.33) | 7 (70) |
| Rhinitis | 3 (75) | 8 (66.67) | 1 (16.67) | 7 (70) |
| Cough | 2 (50) | 4 (33.33) | 1 (16.67) | 3 (30) |
| Dyspnea | 0 (0) | 0 (0) | 0 (0) | 0 (0) |
| Fever | 2 (50) | 8 (66.67) | 3 (50) | 8 (80) |
| Hyposmia and/or ageusia | 1 (25) | 2 (16.67) | 0 (0) | 0 (0) |
| Poor feeding | 1 (1.47) | 2 (3.45) | 0 (0) | 0 (0) |
| Headache | 0 (0) | 9 (75) | 2 (33.33) | 7 (70) |
| Muscular pain | 1 (1.47) | 3 (5.17) | 2 (33.33) | 3 (7.69) |
| Vomiting | 0 (0) | 0 (0) | 0 (0) | 0 (0) |
| Diarrhea | 1 (25) | 1 (8.33) | 1 (16.67) | 0 (0) |
| Abdominal pain | 0 (0) | 0 (0) | 0 (0) | 0 (0) |
| Tachycardia | 0 (0) | 0 (0) | 0 (0) | 1 (10) |
| Skin rush | 2 (50) | 0 (0) | 0 (0) | 0 (0) |
| Conjunctivitis | 1 (25) | 2 (16.67) | 0 (0) | 0 (0) |
| SARS-CoV-2 reinfection, number of symptoms |  |  |  |  |
| 0 | 0 (0) | 0 (0) | 1 (16.67) | 1 (10) |
| 1 | 1 (25) | 2 (16.67) | 1 (16.67) | 1 (10) |
| >1 | 3 (75) | 10 (83.33) | 4 (66.67) | 8 (80) |
| SARS-CoV-2 reinfection, duration of symptoms (day), median (IQR) | 2 (2 - 2) | 2 (2 - 3) | 2 (2 - 2) | 2 (2 - 3) |

§ Clinical management. https://apps.who.int/iris/bitstream/handle/10665/338871/WHO-2019-nCoV-clinical-web_annex-2021.1-eng.pdf (2021).

¥ Participants were classified according to the predominant circulating SARS-CoV-2 VOC in the Veneto Region at the time of children's infection onset (index date) using the CovSPECTRUM platform, that is based on surveillance data [Chen C, Nadeau S, Yared M, et al. CoV-Spectrum: analysis of globally shared SARS-CoV-2 data to identify and characterize new variants. Bioinformatics. 2022;38(6):1735-1737. doi:10.1093/bioinformatics/btab856]. Any SARS-CoV-2 infection that occurred in the Veneto region from February 2020 to December 11, 2021, had a probability greater than 96% to be caused by Parental or Delta variant of concerns; any SARS-CoV-2 infection that occurred in the Veneto region after January 7, 2022 had a probability greater than 96% to be caused by Omicron variant of concerns.
